# From counsel to consumption: examining sociocultural influences on perinatal nutrition in rural India

**DOI:** 10.3389/fnut.2025.1645528

**Published:** 2025-08-07

**Authors:** Faiz A. Hashmi, Cristine H. Legare

**Affiliations:** Center for Applied Cognitive Science, The University of Texas at Austin, Austin, TX, United States

**Keywords:** Accredited Social Health Activist (ASHA), community health workers, cultural dietary beliefs, health behavior change, maternal nutrition, mixed methods research, food taboos, India

## Abstract

Perinatal nutrition is vital for better maternal and child health outcomes. Biomedical guidelines recommend increasing caloric intake, avoiding toxins, and consuming a diverse, nutritious diet during pregnancy and postpartum. Yet, a significant gap persists between nutritional recommendations and actual practices in resource-limited settings. This gap is further complicated by cultural beliefs and traditional practices surrounding food and nutrition, which influence dietary habits. This study examines perinatal dietary practices in rural Bihar, India. We focused on three key areas: caloric adequacy, micronutrient consumption, and food avoidance patterns. We investigated how education and other social determinants influence these dietary choices. We compare dietary behaviors between recent mothers and Accredited Social Health Activists (ASHAs). ASHAs counsel rural mothers on nutrition throughout India and serve as natural benchmarks for assessing whether biomedical recommendations are translated into personal practice. We employed a mixed-methods approach combining qualitative and quantitative methods. Qualitative data included 8 focus group discussions involving 40 young mothers and 38 mothers-in-law, key informant interviews across 6 health influencer categories, and ethnographic observations of 13 ASHAs and 7 households. We conducted a quantitative survey of 400 ASHAs and 1,200 mothers across multiple districts in Bihar. Data were analyzed using descriptive statistics, generalized linear models, and thematic analysis. A significant gap exists between guidelines and actual practices; 85% of mothers and 75% of ASHAs did not increase dietary intake during pregnancy. Education emerged as the strongest predictor of dietary behaviors; women with 14–17 years of education were three times more likely to adopt healthier diets during pregnancy than women with less education. Rice-ghee combinations and milk represented cultural consensus items, while micronutrient-rich foods faced variable acceptance. Notably, more educated women increased overall dietary intake and avoided specific foods, indicating nuanced dietary decision-making rather than simple restriction reduction. Interventions must leverage culturally endorsed foods while promoting incremental modifications to restrictive practices. Educational interventions show promise, given the gradient effect of education on dietary practices. ASHAs serve as crucial cultural brokers but require structural support to overcome community barriers. Addressing maternal undernutrition requires engaging cultural dietary beliefs while enhancing educational opportunities to empower women’s informed dietary choices.

## Introduction

The perinatal period, spanning from conception through the initial weeks postpartum, represents a critical window that significantly influences maternal and child health outcomes across generations (1, 2). Optimal maternal nutrition during this period has been identified as a public health priority, with potential connections to the Sustainable Development Goals and outcomes such as neonatal mortality and morbidity (3, 4). However, translating nutritional recommendations into practice often presents significant challenges, particularly in regions characterized by diverse cultural landscapes and socioeconomic disparities (5). South Asia exemplifies these complexities, where maternal undernutrition may contribute to intergenerational cycles that could perpetuate health inequities and development challenges (1, 6). The implementation of evidence-based dietary guidelines in such settings involves multiple intersecting factors (7). In culturally diverse contexts, such as India, social determinants—including education, caste, access to health information, and traditional dietary beliefs—can shape maternal dietary behaviors (8, 9). Research examining how biomedical nutritional guidelines intersect with culturally rooted dietary practices and social determinants can provide valuable insights for developing contextually appropriate maternal nutrition interventions.

Bihar, a populous eastern Indian state, continues to report among the nation’s highest rates of maternal undernutrition and infant mortality despite substantial investments in maternal and child health programs (10). Pregnant women in Bihar encounter significantly greater nutritional challenges compared to national averages. According to recent National Family Health Survey (NFHS-5) data, they exhibit higher anemia rates (63.1% versus 52.2%), a greater prevalence of below-normal BMI (25.6% versus 18.7%), and lower compliance with iron and folic acid supplementation (18% versus 44%). These figures highlight severe maternal undernutrition and notable gaps in nutritional support efforts (11–14). These deficits contribute directly to low birth weight and stunting, fueling an intergenerational cycle of disadvantage that is difficult to disrupt without targeted, context-sensitive interventions (15). Understanding the translation of nutritional knowledge into practical dietary practices is crucial for enhancing maternal and child health outcomes. In culturally complex settings such as rural Bihar, examining how similar sociocultural contexts produce distinct nutritional behaviors among groups with differing exposure to health education offers valuable insights. Identifying where biomedical recommendations align or diverge from local practices can illuminate practical intervention opportunities that respect and leverage cultural norms.

Biomedical guidelines recommend that pregnant and lactating women progressively increase caloric intake, 350 kcal/day during pregnancy and an additional 450–500 kcal/day during lactation, while consuming a diverse diet rich in protein, iron, folic acid, vitamin A, and other essential micronutrients (16–18). However, adherence to these recommendations remains low in Bihar, highlighting the gap between biomedical guidelines and actual practice (19). While food insecurity and economic constraints undoubtedly contribute to poor dietary practices, previous research in Bihar has revealed that traditional beliefs have a significant influence on perinatal dietary choices (20, 21). Women incorporate nutritious foods, such as milk and pulses, for their health benefits while avoiding other nutritious foods, like wood apples and eggplant, due to fears about miscarriage and birth defects in newborns (19). Critically, family and community members influence dietary choices far more than community health workers, highlighting the influence of kin and the importance of local knowledge systems. Iron-folic acid (IFA) supplementation represents a critical component of perinatal nutrition interventions in India, addressing the high prevalence of anemia among pregnant women (10, 22). Government programs provide free IFA tablets to pregnant women through the health system, including distribution by ASHAs. However, consumption patterns remain suboptimal, with numerous barriers to consistent uptake including side effects, inadequate counseling, and supply chain issues (23, 24).

The gap between nutritional recommendations and dietary practices in Bihar reflects a complex interplay of biomedical guidance and deeply rooted sociocultural beliefs. Traditional food classifications, particularly the concept of “hot” and “cold” foods, profoundly shape dietary choices during pregnancy and postpartum periods. Nutritious “hot” foods such as eggs and pineapple are routinely avoided due to fears of miscarriage, while broader cultural anxieties about fetal overgrowth potentially contributing to birth complications lead many communities to restrict overall food consumption during pregnancy (25–27). These restrictions extend into the postpartum period, where women consume specific foods like ghee while avoiding others deemed “cold,” based on beliefs about maternal recovery and infant health (28). Such culturally prescribed food taboos create a healthcare environment where women must constantly negotiate between evidence-based nutritional guidance and traditional practices (29, 30).

These cultural dietary restrictions intersect with environmental constraints that further compromise nutritional intake. Seasonal variations in food availability create significant fluctuations in dietary diversity throughout the year, with local food systems offering inconsistent access to nutrient-dense foods (31). While research from neighboring states demonstrates that women consuming at least five food groups daily experience fewer pregnancy complications, only 23% of pregnant women in Bihar achieve this dietary diversity benchmark (32). Climate change compounds these challenges by disrupting agricultural yields and forcing households to adopt less varied dietary patterns during the critical periods of pregnancy and lactation (33).

Within this context of cultural beliefs and environmental constraints, women’s agency in dietary decision-making is acritical yet complex factor. Rather than making individual choices, pregnant and lactating women navigate intricate negotiations among personal preferences, household expectations, healthcare recommendations, and economic realities (34). Economic independence significantly enhances women’s nutritional autonomy—those engaged in paid work consume more protein-rich foods during pregnancy, regardless of household income (35). Education similarly strengthens women’s ability to integrate biomedical recommendations with culturally appropriate practices (36). However, traditional household structures often undermine this agency, particularly through the influence of older females (especially mothers-in-law) who typically control food purchasing, preparation, and distribution decisions in joint families (37). As pregnant and lactating women frequently receive lower priority in food distribution, their access to nutrient-dense items remains limited even when available, with mothers-in-law often enforcing cultural food taboos and restrictions based on traditional beliefs (38, 39).

Recognizing these multifaceted challenges, the Indian healthcare system has positioned Accredited Social Health Activists (ASHAs) as crucial intermediaries in improving perinatal nutrition. These community health workers address the knowledge-practice disconnect between biomedical recommendations and actual behaviors while navigating the limited integration of traditional beliefs with scientific guidance. Coming from the communities they serve, ASHAs share cultural backgrounds with their beneficiaries but possess enhanced education and health-related training, enabling them to function as cultural brokers between biomedical and traditional health systems (40, 41). Their role extends beyond providing nutritional information to facilitating access to government services and supplements, while working within existing household decision-making structures to enhance women’s nutritional agency (42–45).

Despite ASHAs’ unique positioning as cultural brokers, implementation science frameworks reveal critical gaps between their professional training and practical effectiveness. The Knowledge-to-Action framework emphasizes that successful implementation involves not only information transfer but also adaptation to local contexts, addressing implementation barriers, and monitoring outcomes—processes that often remain incomplete in Bihar’s nutrition programs (46–48). Social learning theory further suggests that ASHAs’ effectiveness depends significantly on their perceived credibility as legitimate role models within their communities (49, 50). When community members observe discrepancies between ASHAs’ advice and personal practices, the persuasiveness of nutritional messaging can diminish substantially (51–53). Incorporating these implementation dynamics is essential for designing training approaches that enable ASHAs to effectively bridge the gap between nutritional guidelines and actual dietary behaviors.

Accredited Social Health Activists’ personal perinatal dietary practices are crucial beyond their role as health educators. As women embedded in the same cultural context as their beneficiaries, their lived experiences reflect a real-world negotiation between biomedical recommendations and sociocultural constraints (54). Our findings reveal that ASHAs’ personal health behaviors strongly predict their clients’ practices during pregnancy, with ASHAs who adhere to biomedically recommended behaviors like early antenatal checkups, as well as those who engage in traditional practices such as concealing pregnancy or calling traditional midwives, having clients who exhibit similar behaviors (29). The congruence (or lack thereof) between ASHAs’ professional advice and personal practice directly impacts their credibility within communities (7, 55). When ASHAs personally adhere to the dietary practices they recommend, they can serve as authentic role models whose lived experience reinforces their messaging (56, 57). Conversely, discrepancies between knowledge and practice reveal practical barriers to implementation that likely affect their beneficiaries even more significantly (58, 59).

A comparative analysis of ASHAs and their beneficiaries (pregnant and postpartum women from the communities ASHAs serve) offers unique methodological advantages for identifying effective intervention points. Both groups share similar cultural pressures and food availability constraints but differ significantly in their exposure to health knowledge: ASHAs receive formal nutrition training, while mothers primarily rely on traditional knowledge and family guidance (19, 20). This natural comparison enables isolation of the impact of health education versus structural barriers, distinguishing knowledge-based constraints addressable through education from system-level challenges requiring broader interventions (60). Examining whether ASHAs’ professional knowledge translates into personal practice (“practice versus preaching”) can highlight implementation barriers impacting all women and identify practices bridging biomedical recommendations with cultural acceptability (5). Understanding discrepancies between ASHAs’ recommendations and their personal practices is critical for credibility assessment, as these gaps can undermine their effectiveness as role models and reduce counseling impact. While considerable research on maternal nutrition in India exists, studies systematically leveraging this comparative approach to understand social determinants of dietary practices are limited. Such analysis is crucial for prioritizing interventions addressing the most significant barriers to optimal perinatal nutrition in culturally complex regions like Bihar (61, 62).

The primary objective of this study is to investigate how social determinants—including education, caste, religion, and parity—influence perinatal dietary practices in rural Bihar across three critical dimensions: (1) caloric adequacy during pregnancy and postpartum, (2) patterns of food avoidance that may compromise nutrient intake, and (3) diversity in the consumption of foods rich in essential micronutrients, which are critical for maternal wellbeing and fetal development. Our second objective is to compare dietary practices between ASHAs and mothers to understand how formal health training translates into personal practice. This comparison between women who share similar sociocultural backgrounds but differ in healthcare knowledge allows us to identify where biomedical recommendations align with or diverge from local practices, distinguishing between universal implementation barriers and training-specific gaps while illuminating culturally appropriate intervention opportunities (5, 63).

## Materials and methods

### Study setting and design

The study employed a mixed-methods approach to examine and compare perinatal dietary practices among ASHAs and mothers in Bihar, identifying gaps and alignments between dietary beliefs and actual behaviors. Bihar represents an ideal setting for this research as India’s most rural and economically disadvantaged state, where persistent challenges in maternal nutrition exist despite ongoing health initiatives. Our comprehensive research design integrated qualitative, ethnographic, and quantitative components to triangulate findings across multiple data sources.

### Qualitative methods

#### Focus group discussions

##### Participant recruitment

For focus group discussions (FGDs), we purposively selected two distinct participant groups—young mothers and mothers-in-law—in collaboration with local health officials to capture intergenerational perspectives on dietary practices. Participants were recruited from the catchment areas of Anganwadi centers based on specific inclusion criteria: mothers must have given birth within the past 6 months, while mothers-in-law must have a daughter-in-law who had recently given birth. Within these groups, we ensured variation across key demographic characteristics and caste categories.

##### Data collection

Focus group discussions were conducted across 21 villages in Nalanda and Samastipur districts during January 2019. These districts represent Bihar’s sociocultural diversity: Nalanda (South Bihar alluvial plains, Magahi-speaking, rice-wheat cultivation) and Samastipur (North Bihar plains, Maithili-speaking, rice-maize systems). While not representative of all 38 districts, they capture key variations in agricultural systems, linguistic communities, and urban proximity affecting dietary patterns. Forty FGDs were conducted, equally split between younger mothers (births within 2 years) and older mothers (with daughters/daughters-in-law with children under two), segregated by religion for comparative analysis. The groups ranged in size from four to seven participants, with a median of five participants per discussion. Semi-structured discussion guides explored food beliefs, daily dietary practices, pregnancy rituals, and household decision-making dynamics. All FGDs were audio-recorded, transcribed verbatim in Hindi, and then translated into English.

Our analytical approach involved the rapid descriptive coding of each practice/belief statement, followed by inductive thematic grouping to identify patterns across the data (64). This two-stage process allowed initial categorization of discrete practices while remaining open to emergent themes that cut across predetermined categories. To enhance analytical rigor and identify dominant narratives, we conducted frequency tallies by speaker type and district, integrating quantitative counting with qualitative interpretation (65). Two independent coders performed peer debriefing throughout the analysis, with discrepancies resolved through consensus discussion to ensure coding reliability and interpretive validity.

#### Key informant interviews

##### Participant recruitment

Key informant interviews (KIIs) were conducted concurrently with FGDs in the same 21 villages during January 2019. We employed a combination of snowball and convenience sampling to recruit community influencers who shape maternal health decision-making. Key informants encompassed three categories of community influencers: community health workers including ASHAs and Anganwadi Workers (AWWs) identified through local health centers; traditional health providers comprising Rural Medical Practitioners (RMPs) and Dais (traditional birth attendants) identified through community referrals; and religious leaders including Hindu Pandits and Muslim Mulanas recruited through local religious institutions.

##### Data collection

We conducted 50 KIIs across 6 stakeholder categories representing the maternal health ecosystem. Community health workers comprised the largest group, with 12 ASHAs and 11 AWWs. Traditional health providers included 10 Traditional Birth Attendants/Dais and 6 RMPs. Religious leaders consisted of five Hindu Pandits and six Muslim Maulanas, providing crucial insights into faith-based dietary restrictions. Interview guides were designed to parallel FGD prompts, enabling systematic comparison across data collection methods and triangulation of findings regarding maternal dietary practices and health-seeking behaviors. Interview guides paralleled FGD prompts to enable cross-method comparison.

All interviews were recorded, transcribed verbatim, and translated from Hindi/local dialects by bilingual research assistants. Analysis combined deductive and inductive approaches (66), with initial rapid coding using predetermined domains (dietary practices, health beliefs, and service delivery) while remaining open to emergent themes. Domain grouping organized coded segments into higher-level categories to reveal patterns across stakeholder groups. Frequency counts identified recurring themes by informant type, quantifying which issues were most salient for different groups, enhancing pattern recognition while preserving context (65). Strategic quote selection balanced representative responses with unique insights that revealed exceptional cases.

#### Ethnographic component

##### Participant recruitment

For the ethnographic fieldwork, ASHAs were selected using criterion-based sampling focused on diversity in years of service and village settings (remote versus accessible). Selection required only that ASHAs be actively serving in their communities; their personal maternal status was not a consideration, as observations centered on professional practices rather than personal dietary behaviors. Participating households were selected based on having either a pregnant woman or a lactating mother with an infant under 6 months, ensuring opportunities to observe dietary practices during these critical periods.

##### Data collection

We conducted focused ethnographic fieldwork in the Samastipur district from February to March 2019. This 6-week intensive period was strategically timed to coincide with key maternal health events. The field team shadowed 13 ASHAs across four blocks, observing them in multiple contexts: home visits during HBNC, community health meetings at Anganwadi centers, immunization sessions, two Primary Health Center family-planning days, and informal village interactions. Observations documented nutrition-related discussions as part of maternal and newborn care, including dietary advice provided, family members’ responses to counseling, and ASHAs’ handling of conflicts between biomedical recommendations and local beliefs. These observations were supplemented by opportunistic informal interviews with an ASHA trainer, two Auxiliary Nurse Midwives, one traditional birth attendant, and multiple family members present during visits. All observations and informal conversations were documented through detailed field notes written within 24 h of each session.

Data were recorded as detailed narrative field notes and thematically coded according to three dietary domains plus service-delivery context. Caloric adequacy encompassed observations of whether women increased food quantity during pregnancy/postpartum as recommended, including family discussions about eating “more than usual” versus maintaining or reducing intake. Food avoidance documented cultural restrictions and taboos. Micronutrient intake captured observations of women adding nutrient-rich foods (green leafy vegetables, fruits, dairy, and meat) or taking supplements like IFA, as well as barriers to accessing these items. This ethnographic component provided rich, contextual insights into how nutritional recommendations translate into actual practices within household dynamics and cultural constraints.

#### Quantitative survey

##### Participant recruitment

For the quantitative survey, we implemented a multi-stage cluster random sampling across Bihar’s three major linguistic regions: Maithili (Samastipur and Purnia districts), Magahi (Gaya district), and Bhojpuri (West Champaran district). In the first stage, two blocks were randomly selected from four districts using probability proportional to size methodology. The second stage involved random selection of 50 Anganwadi centers from each selected block, utilizing the complete list of centers maintained by the Integrated Child Development Services (ICDS) program. These Anganwadi centers served as primary sampling units. In the final stage, households with eligible women (pregnant women and mothers with infants under 6 months) were identified through Anganwadi registers and selected using systematic random sampling, with every fifth household chosen based on the required sample size per center.

##### Data collection

The sample size was determined based on detecting differences in dietary practices between ASHAs and mothers (Table 1). Assuming 50% of mothers follow recommended dietary practices (based on prior studies on maternal diets in India), we needed 364 participants per group to identify a minimum difference of 10% points with 80% power and a 5% significance level. Accounting for a 10% non-response rate, we aimed to recruit 400 participants per group. The final sample included 400 ASHAs and 1,200 mothers, providing sufficient power for subgroup analyses by education and caste. The 1:3 ratio maximized resource use while maintaining statistical strength for comparisons.

**TABLE 1:** Demographic and socioeconomic characteristics of participating mothers and ASHAs.

		Mothers		ASHAS	
Variable	Level	*N*	%	*N*	%
Religion	Hindu	1,049	87.4	386	96.5
	Muslim	143	11.9	12	3.0
	Other	8	0.7	2	0.5
Caste	General	80	6.7	68	17.0
	Scheduled caste	372	31.0	67	16.8
	Scheduled tribe	18	1.5	3	0.8
	OBC^1^	730	60.8	262	65.5
Education^2^	0	592	49.3	3	0.8
	1–6	107	8.9	1	0.3
	7–8	130	10.8	88	22.0
	9–10	199	16.6	179	44.8
	11–13	100	8.3	91	22.8
	14–17	72	6.0	38	9.5
Parity^3^	1	336	28.0	21	5.3
	2	320	26.7	77	19.3
	3	287	23.9	148	37.0
	4	159	13.3	94	23.5
	5+	98	8.2	60	15.0

^1^OBC, other backward classes.

^2^Education categories: 0 = no formal education/illiterate; 1–6 = primary education; 7–8 = middle school; 9–10 = high school; 11–13 = higher secondary; 14–17 = graduate and above.

^3^Parity = total number of live births.

Sample characteristics included ASHAs with 1–20 years of service experience (median: 12 years), comprising those who had experienced childbirth before becoming ASHAs (73%) and during their service (27%). All mother respondents had given birth within 6 months before data collection to minimize recall bias. The sample captured demographic diversity typical of the study area: mothers aged 15–41 years (mean: 23) and ASHAs aged 23–65 years (mean: 38); educational levels ranging from non-literate mothers (approximately 50%) to ASHAs with mandatory minimum 8 years of education; and religious composition reflecting local demographics (mothers: 87.4% Hindu, 11.9% Muslim, and 0.7% other; ASHAs: 96.5% Hindu, 3% Muslim, and 0.5% other).

The survey instrument explored perinatal dietary practices during pregnancy and postpartum periods, collecting data on dietary changes, specific food items added or avoided, influential actors, and motivating reasons. An extensive list of food items was generated through FGDs and KIIs to inform the development of the survey. Participants were asked about their consumption of various food items that they added to or avoided during their pregnancy and postpartum periods. These food items were subsequently categorized into various food groups during the post-coding analysis phase. The survey also gathered information on complementary health behaviors and demographic characteristics, including IFA supplementation practices.

### Instrument development and validation

We developed culturally appropriate instruments through a rigorous multi-stage process. Initial English instruments were constructed from validated tools, including the NFHS-5 and WHO maternal nutrition assessment guidelines. Bilingual research staff at PCI India translated instruments into Hindi and Maithili, with independent back-translation conducted to verify semantic equivalence. We pilot-tested the survey with mothers and ASHAs in a non-study village, making minor wording adjustments based on feedback to enhance local dialect comprehension. This comprehensive validation process ensured instruments maintained scientific rigor while remaining culturally accessible to our target population.

### Quality assurance and bias mitigation

We implemented several strategic measures to enhance data quality and minimize bias. To combat recall bias, we deliberately recruited mothers within 6 months of delivery—a timeframe when pregnancy experiences remain vivid—and anchored our structured interviews to memorable pregnancy milestones while incorporating visual food aids to prompt accurate recollection. Recognizing that dietary practices often carry cultural judgment, we carefully addressed social desirability bias by ensuring interviews were conducted privately by highly experienced female field investigators from the same regional background as participants. These seasoned researchers, well-versed in local cultural nuances, established rapport while providing explicit assurances of anonymity. Our dietary assessment framework was informed by insights from prior qualitative data synthesis, ensuring cultural relevance and comprehensiveness. To address potential role conflict, ASHA participants were explicitly instructed to discuss their dietary practices and beliefs as mothers and community members rather than in their professional capacity. Personal dietary experiences were explored before professional practices to reduce social desirability bias, with all ASHAs interviewed individually to encourage authentic sharing of personal information.

### Data analysis

We employed a mixed analytical approach appropriate to each data type. For quantitative data, we calculated descriptive statistics (frequencies and percentages) and developed generalized linear models using logistic regression for binary outcomes (e.g., whether dietary intake increased) and negative binomial regression for count data (e.g., number of foods avoided) after confirming overdispersion relative to the Poisson distribution.

Models maintained consistent covariates, including caste, religion, education, parity, and age as predictors. We fitted separate models for pregnancy and postpartum periods. To ensure model validity, we calculated variance inflation factors, confirming no independent variables exceeded values of 4.0, indicating minimal multicollinearity. The analysis emphasized odds ratios with 95% confidence intervals (CIs), excluding 1.0 as statistically significant, while noting trends in effect sizes across variable levels.

All quantitative analyses were conducted using R (version 4.03), incorporating packages including tidyverse, MASS, broom, car, and Hmisc. Qualitative and ethnographic data underwent thematic analysis to identify patterns of convergence and divergence with quantitative findings, enabling robust triangulation across methods. Detailed descriptions of qualitative findings are provided in the supplementary data sheets: ethnographic observations (Supplementary Data Sheet 1), focus group discussions (Supplementary Data Sheet 2), and key informant interviews (Supplementary Data Sheet 3).

## Results

This section synthesizes findings from four complementary methodological approaches: a quantitative survey of ASHAs and mothers, FGDs with young mothers and mothers-in-law, KIIs with diverse stakeholder cadres, and focused ethnographic observations. We present quantitative patterns first to establish prevalence and statistical relationships, then integrate qualitative findings to illuminate cultural contexts and decision-making processes. This mixed-methods triangulation examines perinatal dietary practices across three critical domains: caloric adequacy, micronutrient consumption, and food avoidance patterns.

### Caloric adequacy during pregnancy and postpartum

#### Qualitative insights

##### Primary staples and energy-dense foods

Qualitative data illuminated the cultural underpinnings of caloric intake patterns. The FGDs revealed that rice and khichri dominate meals, with elders framing rice as “heavier” than wheat, especially in the third trimester. As one mother-in-law explained: “*Rice keeps the stomach full; without it, the mother feels empty.*” A young mother reported: “*I asked for roti but they said only rice gives ‘weight’ to the baby.*”

This rice-centric diet was consistently reported across data streams. KIIs confirmed that rice or khichdi constitutes the baseline meal, with Dais and ASHAs urging an extra serving in the third trimester. An AWW stated: “*After delivery, Mother is given Bread, Biscuit, Milk, Rice, and Vegies to eat*,” while an ASHA reported: “*Fish, meat, pulses, and rice are cooked, and mother takes this food by taking child on lap.*”

For energy density, ghee emerged as the culturally sanctioned caloric booster. FGDs found that affordability, rather than acceptability, limits the use of ghee. A mother-in-law emphatically stated: “*One spoon of ghee in every plate—doctor or no doctor*,” while a young mother noted: “*If ghee is too costly, we use mustard oil, but elders frown.*” KIIs confirmed that ghee remains the preferred calorie booster, with AWWs promoting it, while RMPs noted cost barriers. A Dai simply recommended*:* “*Ghee with bread*,” while another explained: “*Halwa made up of Rice, Ginger, Turmeric, Ghee and jaggery is given to mother so that her body remains warm.*”

##### Postpartum caloric patterns

Ethnographic observations revealed a distinctive postpartum dietary pattern where sweet, energy-dense foods dominate the first postpartum week, while grains and savory items are deferred until the chhathi ceremony (sixth day). A new mother explained: “*I am on a diet of milk and ginger halwa… I cannot have any grains before the sixth day.*” An ASHA, recounting her own deliveries, confirmed: “*Women were given sweets (halwa) and milk for the next six days.*”

Ethnographers also observed that hospital meals (rice, dal, and chicken) are routinely refused as “heavy” or ritually impure. KIIs identified additional hydration strategies, noting that leftover rice water (“*maad*” in Hindi) and sweet tea emerge as low-cost energy drinks, mainly recommended by Dais.

#### Quantitative insights

##### Patterns of overall caloric intake

Among participants who reduced their dietary intake, taste aversion and digestive issues were the dominant reasons for both groups (Table 2). Nearly half cited taste issues (47.2% of mothers, 48.1% of ASHAs) and digestive problems (44.3% of mothers, 47.5% of ASHAs). Mothers were more likely than ASHAs to report pressure on the womb as a reason for eating less (19.8% versus 15.5%). Concerns about difficulty in labor were cited by only 4% of mothers and 2.2% of ASHAs who reduced intake. Among those who increased their dietary intake, motivations differed between mothers and ASHAs (Table 3). Maternal health was the primary reason for both groups (72.8% of mothers, 83.7% of ASHAs), followed by newborn health (60% of mothers, 80.8% of ASHAs). ASHAs were more likely than mothers to cite proper fetal growth (20.2% versus 10.6%) and ease of labor (20.2% versus 11.7%) as reasons for increasing intake. Notably, some mothers cited taste preferences (16.1%) as a reason for eating more, while this motivated few ASHAs (4.8%).

**TABLE 2:** Reasons explaining dietary reduction.

	Mothers		ASHAs	
Variable	*N*	Percent	*N*	Percent
Taste	310	47.2	87	48.1
Digestive issues	291	44.3	86	47.5
Pressure on the womb	130	19.8	28	15.5
Other	70	10.7	32	18.2
Maternal illness	68	10.4	23	12.7
Difficulty in labor	26	4	4	2.2
Newborn deformities	8	1.2	4	2.2
Newborn illness	7	1.1	3	1.7
Do not know	5	0.8	0	0
Miscarriage	0	0	1	0.6

**TABLE 3:** Reasons explaining dietary increase.

	Mothers		ASHAs	
Variable	*N*	Percent	*N*	Percent
Maternal health	131	72.8	87	83.7
Newborn health	108	60	84	80.8
Taste	29	16.1	5	4.8
Ease labor	21	11.7	21	20.2
Proper fetal growth	19	10.6	21	20.2
Other	16	8.9	5	4.8
To prevent illness	10	5.6	16	15.4
Do not know	4	2.2	0	0
Newborn beauty	3	1.7	0	0

**FIGURE 1:**
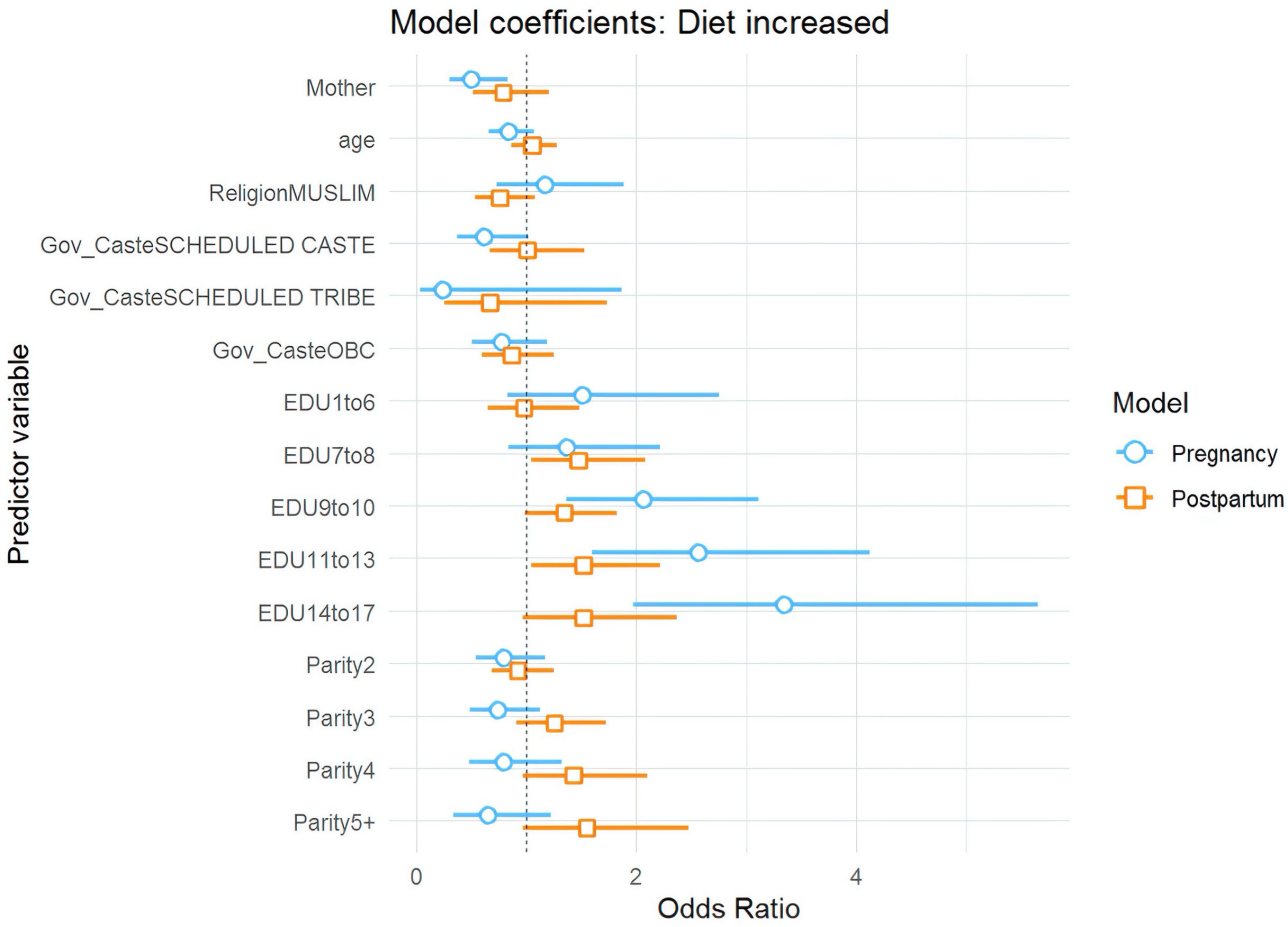
Plot of regression coefficients for increased dietary intake during pregnancy and postpartum.

The survey identified several factors associated with dietary changes. Being a mother (versus an ASHA) and being a member of a Scheduled Caste (versus General Caste) were negatively associated with increasing diet during pregnancy (Figure 1). Mothers were about 51% less likely than ASHAs to increase their diets (OR = 0.49, 95% CI: 0.29–0.83), while members of Scheduled Castes were about 39% less likely than General Caste to do so (OR = 0.61, 95% CI: 0.37–1.02).

**FIGURE 2:**
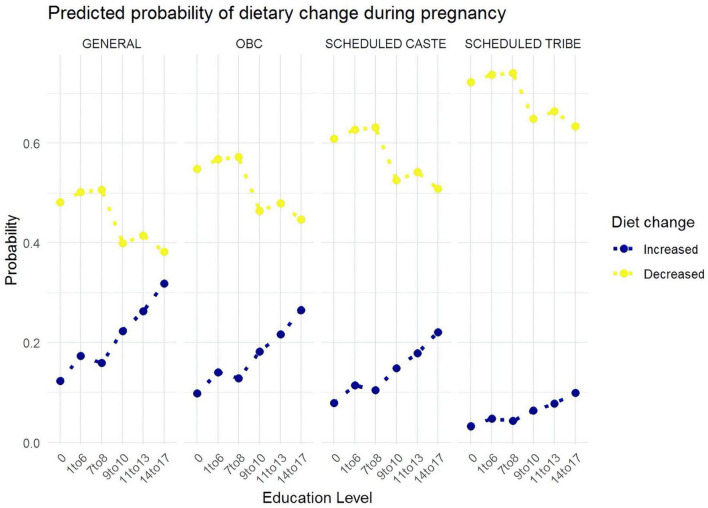
Predicted probabilities of increasing dietary intake and decreasing dietary intake during pregnancy.

Education emerged as a consistently strong predictor (Figures 1, 2). As educational attainment increased, so did the likelihood of increasing diet: women with 9–10 years of schooling were twice as likely to increase their dietary intake as uneducated women (OR = 2.06, 95% CI: 1.36–3.12), while those with 14–17 years of education showed even greater improvements, being more than three times as likely to increase their diets (OR = 3.34, 95% CI: 1.97–5.64). Postpartum, education continued to show an effect, though weaker than during pregnancy, with women having 7–8 years of education being about 47% more likely to increase their diets (OR = 1.47, 95% CI: 1.04–2.08) compared to those with no education.

### Patterns of food avoidance that may compromise nutrient intake

#### Qualitative insights

##### Cultural taboos and food restrictions

Qualitative data revealed multiple categories of food avoidance linked to cultural beliefs. FGDs highlighted taste and temperature taboos, where sour and “hot” foods were feared to cause miscarriage or body heat, with this rule enforced by elders. A mother-in-law warned: “*Sour cuts the womb like a blade—better stay safe*,” while a young mother recalled: “*I craved mango pickle, but mother said ‘too hot.*’”

Color taboos were also identified, with black-colored foods linked to fears of a dark or weak baby. As one mother-in-law stated, “*Black food makes the baby black and weak.*”

Ritual fasting during eclipses emerged as a particularly strict taboo. FGDs found that during a solar eclipse, no food is prepared or eaten, with mothers-in-law imposing strict compliance. A mother-in-law asserted, “*During the eclipse, even a drop of water can curse the child*,” while a young mother confessed, “*I hid a biscuit but felt guilty.*” KIIs confirmed that religious leaders emphasize eclipse fasting, though ASHAs offer mitigation advice (ORS, meal timing). A Dai described: “*During a Solar eclipse, a thread/wood equivalent to the height of the pregnant woman is hung on the wall.*”

##### Salt restriction and other postpartum taboos

Salt restriction emerged as a consistent postpartum practice. FGDs found that salt is withheld for the first 5–6 days postpartum to “purify milk.” A young mother explained: “*Till Chhathi, my tongue forgets salt; only then milk is sweet.*” Ethnographic observations confirmed this, noting that a new mother does not eat salt, as stated by a village elder*:* “*A new mother does not eat salt… cannot touch the hand-pump for 42 days.*”

Mobility restrictions were also common. Ethnographic data showed that mothers “*will stay in that area till the sixth day and will be fed only milk and sweets*,” according to a maternal grandmother. KIIs reported that all cadres reiterate no traveling or heavy lifting late in pregnancy, citing the risk of miscarriage. An RMP advised: “*Women should avoid traveling*,” while an ASHA similarly stated: “*Women should avoid going out of the house and traveling.*”

Contact taboos were also identified, with KIIs revealing that avoiding funerals/dead bodies to ward off “evil influence” is endorsed across groups. An AWW cautioned: “*Pregnant women should avoid looking at dead bodies/funerals.*”

#### Quantitative insights

##### Patterns of food avoidance

**FIGURE 3:**
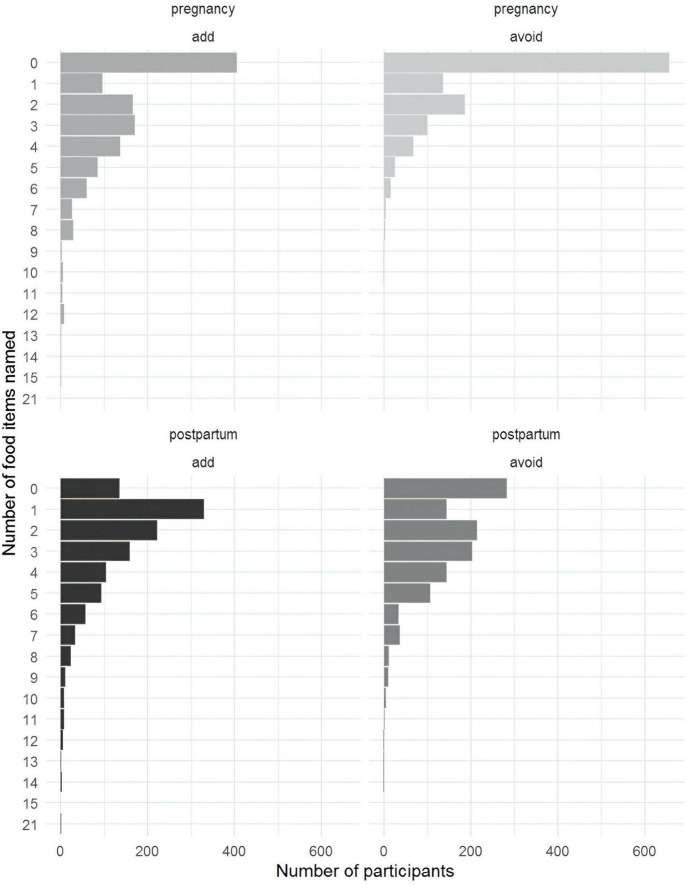
Bar plot of the number of item names by individual respondents per sample (mother data).

**FIGURE 4:**
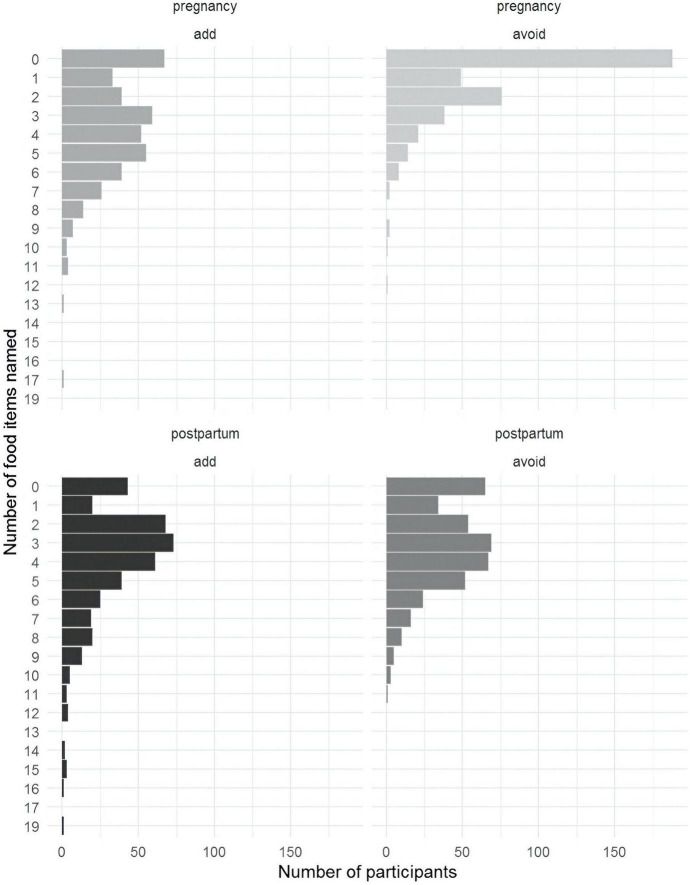
Bar plot of the number of item names by individual respondents per sample (ASHA data).

The quantitative survey examined food avoidance patterns during pregnancy and postpartum. Over half of the mothers did not name any foods to avoid during pregnancy (Figure 3). Postpartum, the most common responses were one food to add and two to avoid. ASHAs tended to name more foods than mothers in general (Figure 4).

**FIGURE 5:**
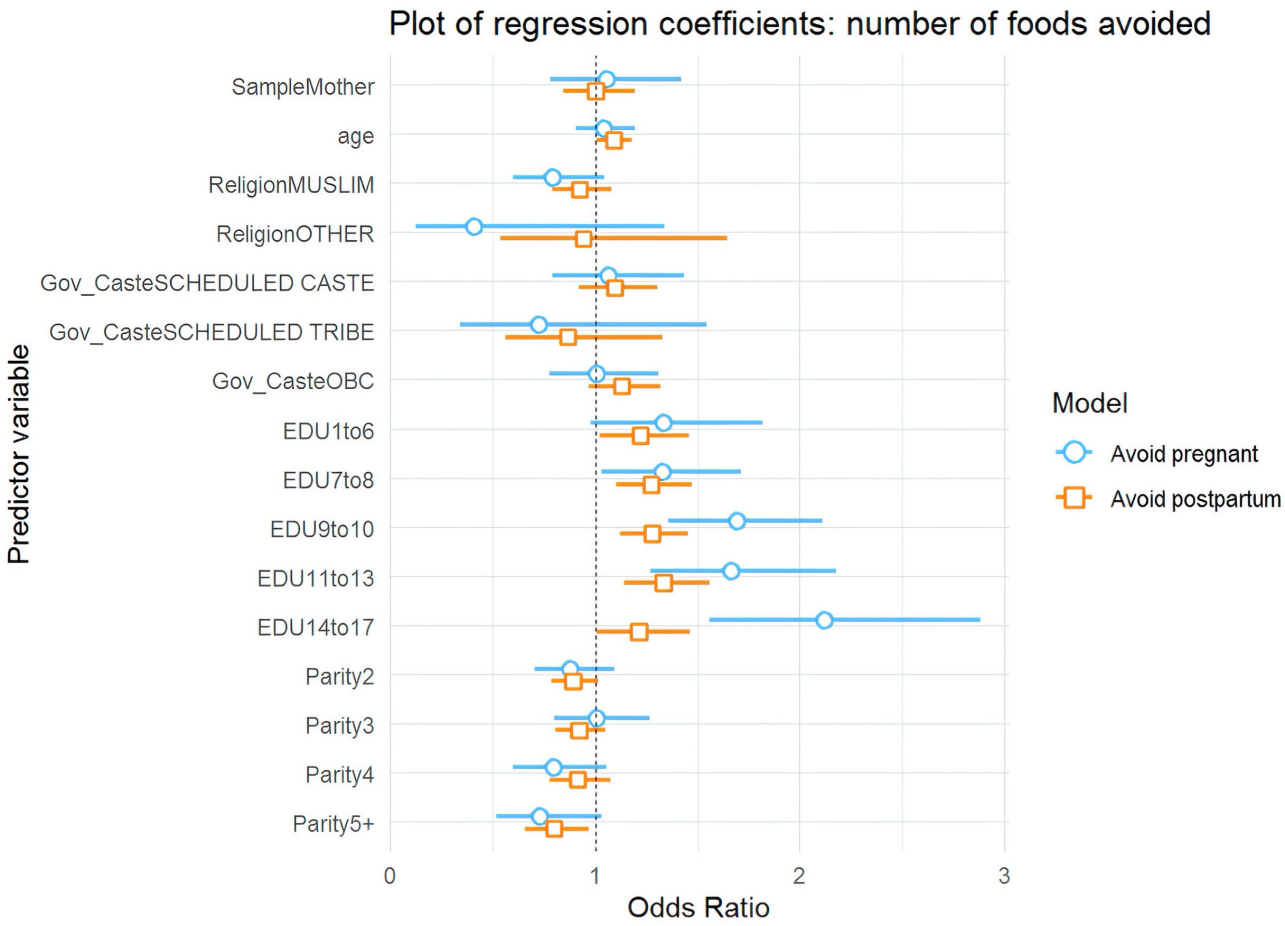
Plot of relative ratios from a negative binomial model for the number of foods avoided during pregnancy and postpartum.

Education again emerged as a key predictor of food avoidance behaviors (Figure 5). Women with 7–8 years of education were expected to avoid about 30% more foods during pregnancy than those with no education (RR = 1.33, 95% CI: 1.03–1.72), and those with 14–17 years of education were expected to name twice as many (RR = 2.12, 95% CI: 1.56–2.89). A similar pattern emerged postpartum, with women with 7–8 years of education expected to avoid about 30% more foods than those with no education (RR = 1.27, 95% CI: 1.10–1.47).

**FIGURE 6:**
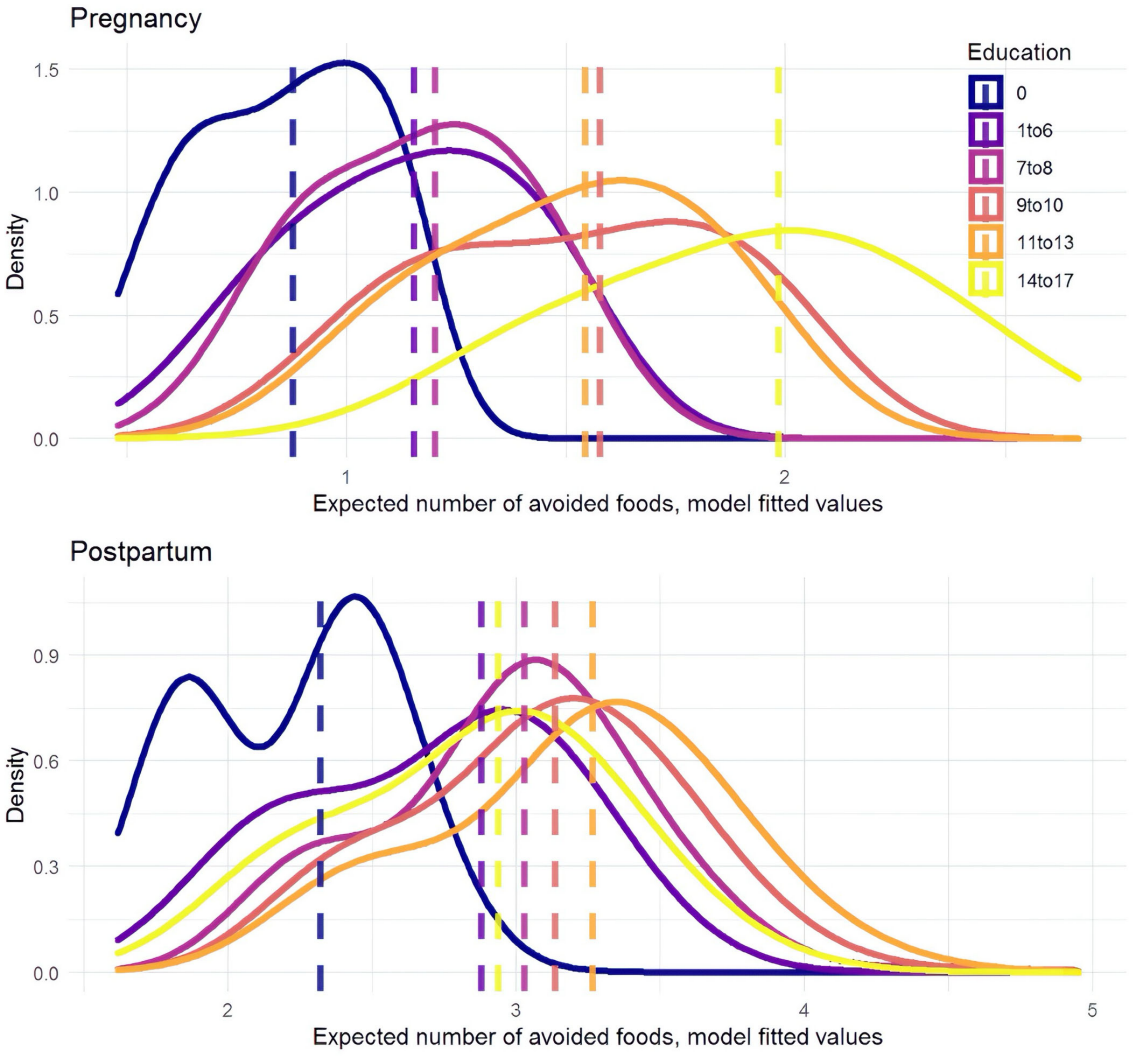
Model fitted values predict the number of foods avoided by education level during pregnancy (upper) and postpartum (lower). More avoided food items are named at higher levels of education.

The quantitative findings presented a seemingly counterintuitive result: more educated women were both more likely to increase their perinatal diets and likely to avoid a larger number of individual foods. We plotted the fitted values for each level of education to further visualize the relationship, which clearly shows the sequentially increasing mean of foods named from each education level to the next (Figure 6). One potential explanation is that education and wealth are highly correlated, giving more educated women more food options. Furthermore, more educated mothers are likely to avoid more food items associated with adverse outcomes compared to less educated women.

### Diversity in consuming micronutrient-dense foods crucial for maternal and fetal wellbeing

#### Qualitative insights

##### Dairy and plant-based micronutrients

Qualitative data revealed strong cultural support for certain micronutrient sources. FGDs found that milk is universally praised, with buffalo milk preferred where available. A mother-in-law claimed: “*A glass of buffalo milk makes bones solid like bamboo.*” KIIs confirmed that milk tops every informant list, with bananas ranked second as an easy, “cool” snack.

For fruits and natural drinks, FGDs revealed that banana and coconut water are seen as cooling and digestion-friendly, with supply differences visible by district. A young mother noted: “*Two bananas settle the stomach, and baby sleeps*,” while a mother-in-law explained: “*Coconut water cools the ‘fire’ inside.*” District-specific patterns were observed, with Nalanda FGDs mentioning fish and coconut water twice as often as Samastipur (reflecting riverine markets). At the same time, Samastipur groups referenced eggs and bananas more frequently (mirroring local poultry and orchards).

For green, leafy vegetables, FGDs noted low spontaneous mention, with prep time cited as a barrier. A young mother explained: “*Spinach is good, but who has time to clean so many leaves?*” KIIs found that spinach and IFA tablets are championed by frontline workers, while elders mention greens without dosage clarity. An ASHA mentioned: “*BP test, TT, Weight, IFA on AWC*,” while a Dai simply stated: “*Spinach.*”

##### Animal protein sources

Animal protein consumption showed interesting patterns across different sources. FGDs found that eggs gain traction with young mothers, though elders are split over “heat” concerns. Fish and meat are promoted by elders when they find it affordable. A young mother confided: “*Doctor says one egg a day, so I eat when nobody sees*,” while a mother-in-law asserted: “*Fish grows the baby’s brain—river gift from God.*”

Key informant interviews revealed that egg promotion is led by ASHAs, while fish and meat are endorsed by Maulanas and Pandits post-delivery. A Maulana described a ritual: “*AKIKA should be performed after 6 days of delivery. In the case of a boy child, two goats’ meat should be distributed. In case of a girl child, one goat’s meat should be distributed.*”

Ethnographic observations, however, noted that while milk and bananas remain acceptable, eggs are absent from the diets of observed women.

##### Iron-folic acid supplementation

Iron supplementation faced significant barriers. Ethnographic observations revealed that IFA tablets are sometimes thrown away due to nausea or concerns about expiry, with one pregnant woman admitting: “*I threw away all the IFA tablets – they made me nauseous.”* Another 5-month-pregnant woman explained: “*I was given tablets, but they were past expiry; I haven’t started yet.*” Ethnographers also noted that expired or mistrusted supplements and a lack of ANM follow-up erode coverage.

##### Breastfeeding and pre-lacteal feeding

Pre-lacteal feeding practices emerged as a significant issue affecting infant nutrition. Ethnographic observations revealed that religious timing often overrides the timely initiation of breastfeeding despite ASHA counseling. A Dai’s husband explained: “*The child is fed goat’s milk until the pandit gives the exact time to start breastfeeding.*” A Senior ASHA confirmed: “*Newborns were given water or cow’s milk until a priest was consulted.*”

Focus group discussions found that elders routinely add water to breastfeed in hot months despite exclusive breastfeeding messaging. A mother-in-law justified: “*In the June heat, the child needs water along with milk, else he burns*,” while a young mother admitted: “*ASHA says no water, but the baby cries; I give a spoon.*”

Key informant interviews revealed a concerning practice where colostrum is discarded, as a Dai stated: “*Colostrum is thrown and then mother’s milk is fed to the child.*”

##### Generational power dynamics and negotiation

Generational power dynamics strongly influence dietary practices. Data from FGDs revealed that mothers-in-law act as gatekeepers of taboos and food distribution. Young mothers comply publicly but adopt hidden strategies (e.g., eating eggs privately) to integrate biomedical advice. Negotiation—not confrontation—is their coping mechanism.

The KIIs revealed both areas of convergence and points of contention across different stakeholder groups. There was broad agreement among stakeholders on the nutritional value of combinations such as rice or khichdi with ghee, as well as the consumption of milk and bananas. In contrast, the inclusion of eggs in the maternal diet and the regular intake of IFA tablets remained contested, reflecting ongoing debates and varied perceptions regarding their appropriateness and benefits during pregnancy. ASHA and AWW narratives emphasize biomedical supplements, while religious leaders frame fish/meat as divinely sanctioned. Mobility and eclipse taboos persist despite ASHA mitigation advice.

#### Quantitative insights

##### Patterns of micronutrient consumption

The quantitative survey examined the consumption of key micronutrient-rich foods and supplements. For leafy green vegetables, mothers self-reported adding them to their diet at lower rates (8.2% during pregnancy and 4.2% postpartum) compared to ASHAs (34.2% during pregnancy and 16.0% postpartum). Similarly, for vitamin A rich foods such as carrots, sweet potatoes, dark leafy greens, and mangoes, mothers reported lower addition rates (3.2% during pregnancy and 0.2% postpartum) than ASHAs (8.8% during pregnancy and 1.8% postpartum).

**FIGURE 7:**
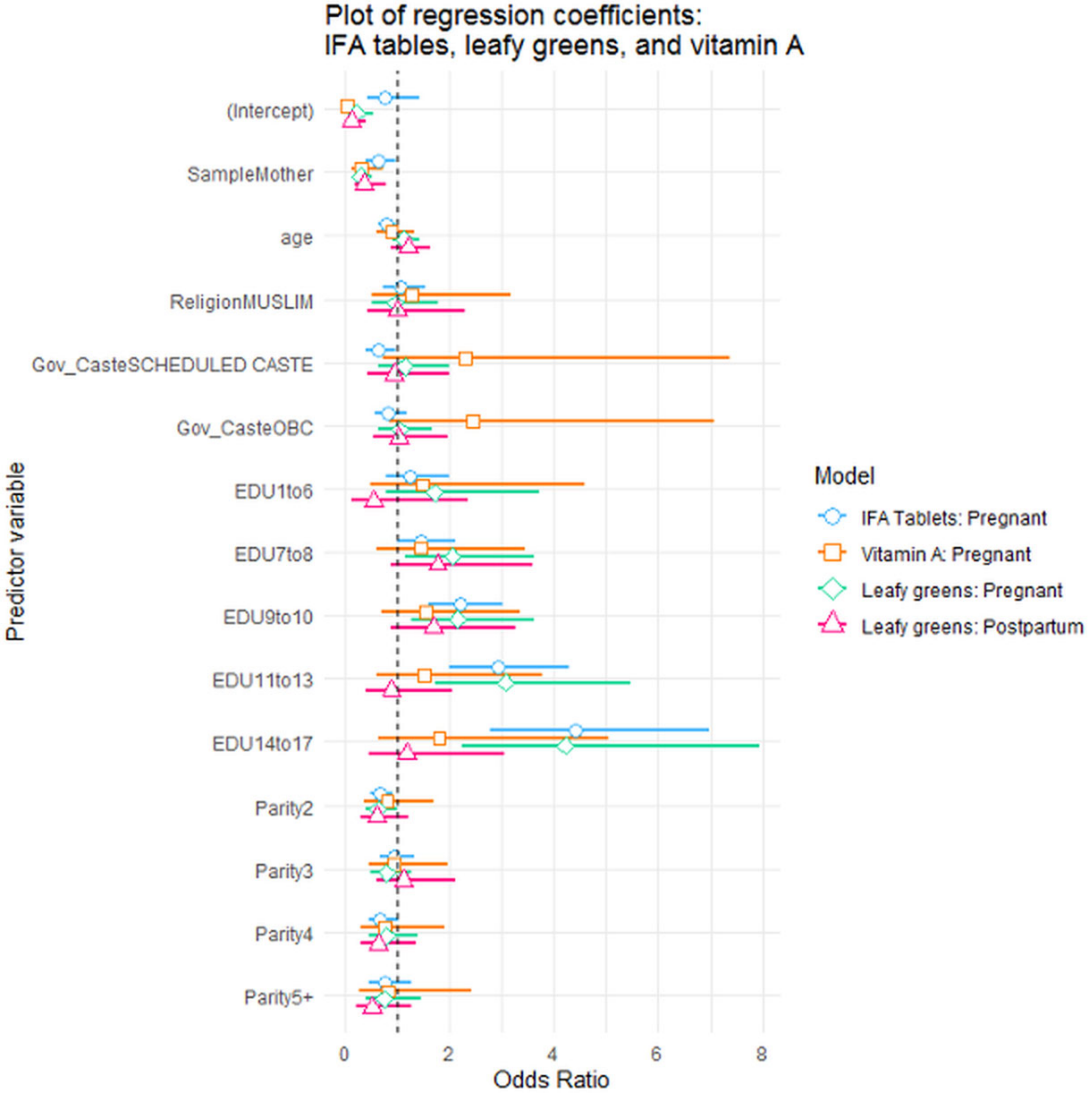
Plot of binary logistic regression coefficients: added leafy greens, vitamin A, IFA tablets.

Statistical analysis identified key predictors of micronutrient consumption (Figure 7). For adding leafy greens during pregnancy, mothers were about 70% less likely than ASHAs to report doing so (OR = 0.30, 95% CI: 0.17–0.51). Education again showed a strong positive effect, with women having 7–8 years of education being about twice as likely to add leafy greens (OR = 2.06, 95% CI: 1.16–3.64) compared to those with no education, and those with 14+ years being over four times more likely.

Consumption of IFA supplements showed a similar pattern, with 34% of mothers reporting taking the complete regimen during pregnancy, compared to 45% of ASHAs. Mothers were approximately 37% less likely than ASHAs to have taken the recommended allowance (OR = 0.63, 95% CI: 0.40–0.97). Women from Scheduled Castes were about 38% less likely than women from General Caste to have taken them (OR = 0.62, 95% CI: 0.41–0.96). Education showed a strong positive effect, with women having 14–17 years of education being over four times more likely to have taken the recommended dosage (OR = 4.41, 95% CI: 2.79–7.03) compared to those with no education.

## Discussion

Our mixed-methods findings reveal how social determinants—particularly education and caste—influence perinatal dietary practices in rural Bihar across three dimensions: caloric adequacy, micronutrient consumption, and food avoidance patterns. By comparing ASHAs and mothers who share cultural backgrounds but differ in health training, we distinguish between knowledge-based barriers and structural constraints that influence nutritional practices. ASHAs serve as a reference for assessing whether health knowledge translates into practice amid shared structural constraints. We also discuss strategies for enhancing ASHA effectiveness through targeted training, cultural negotiation skills, and structural support systems that address barriers they face as both health educators and community members.

### Caloric adequacy: knowledge-practice gap and generational dynamics

Our findings, based on self-reported changes in food quantity rather than measured caloric intake, suggest a significant gap between biomedical recommendations and actual practices. Despite guidelines for progressive caloric increases during pregnancy and postpartum (16, 18), the majority of both mothers and ASHAs reported maintaining or reducing their food intake. While these self-reported patterns cannot confirm actual caloric inadequacy, they indicate potential gaps between recommended and actual behaviors previously noted in the literature (19). Beyond knowledge, our results suggest that caloric intake patterns reflect deeply embedded cultural beliefs. The rice-centric diet, fortified with ghee, represents a cultural consensus that spans generations and stakeholder groups. Similarly, the postpartum transition from sweet, energy-dense foods to more diverse meals after the Chhathi ceremony demonstrates how ritual timing structures nutrition more powerfully than biomedical advice. This aligns with previous observations about how household food systems shape maternal access to nutrients (37).

The powerful effect of education across dietary behaviors suggests that enhancing women’s general education, not just health literacy offers a sustainable path to improved nutrition outcomes. Education appears to facilitate women’s ability to navigate between cultural expectations and health optimization, supporting previous research findings that education enhances women’s nutritional agency beyond simply increasing knowledge (35).

#### Recommendations for caloric adequacy

Nutrition programming should build on the culturally sanctioned rice-ghee foundation by promoting incremental additions rather than wholesale dietary changes. Co-designing postpartum meal plans that pivot from day-6 rituals toward gradual inclusion of diverse nutrients respects cultural timing while addressing nutritional gaps. The Anna Purna Scheme demonstrates how customization for local preferences can enhance acceptance. Programs could develop energy-dense recipes incorporating traditional ingredients, such as ghee, while meeting nutritional standards (67). For hospital and institutional settings, offering culturally acceptable energy-dense options could improve intake during facility stays, where standard meals are frequently refused as “heavy” or ritually inappropriate.

Educational interventions should target mothers-in-law as “guardians of tradition-plus-science” nutrition, acknowledging their authority while introducing modifications to restrictive practices. The Mitanin program’s success in empowering village-level women as health change agents suggests that engaging respected community women, including mothers-in-law, can bridge traditional practices with improved nutrition (68). Positioning ghee-fortified foods as beneficial for both maternal recovery and infant health could leverage existing cultural beliefs while promoting optimal nutrition.

### Food avoidance: negotiating cultural taboos and nutritional needs

The pattern of food avoidance practices reveals complex interactions between traditional beliefs and biomedical recommendations. The counterintuitive finding that more educated women both increased their diets and avoided more specific foods suggests sophisticated dietary decision-making rather than simple restriction reduction. This challenges simplistic views of food taboos as purely harmful and indicates that selective food avoidance may coexist with adequate nutrition when overall dietary diversity is maintained.

Complex cultural food classification systems have a more substantial influence on dietary choices than biomedical advice alone. Taste and temperature beliefs particularly shape these decisions; for instance, women avoid sour foods like tamarind or citrus during pregnancy, believing “sourness cuts the womb,” causing miscarriage. Foods are labeled “hot” or “cold” based on traditional ideas of bodily balance, rather than their actual temperature. “Hot” foods (e.g., eggs and papaya) are believed to harm the baby by overheating the body, while “cold” foods are considered safer (25). These practices persist across India; studies in South India reported nearly universal adherence to restrictive hot/cold diets without scientific basis (69), and similar beliefs in Karnataka led to avoidance of nutritious fruits like papaya and mango, linked to fears of the “evil eye” and effects on the baby’s complexion (70). These classifications operate alongside color associations (dark foods darken the baby), eclipse restrictions (complete fasting prevents deformity), and postpartum salt limitations (ensure pure breastmilk). The district-specific patterns in food mentions illustrate how local food systems create variable access to diverse foods, with significant implications for maternal health outcomes (31).

Young mothers’ strategic negotiation of household power dynamics—complying publicly while finding hidden opportunities to incorporate recommended foods—reveals both constraints on women’s agency and creative adaptations. This aligns with previous observations regarding mothers-in-law as gatekeepers of perinatal dietary practices (38, 39).

Although we did not directly interview male household members, their influence was prominent in women’s narratives, shaping dietary decisions through economic control. Husbands’ gatekeeping of food purchases limited women’s dietary choices, creating a paradox where mothers held theoretical agency in food preparation but lacked practical control over ingredient availability. Additionally, mothers-in-law indirectly reinforced patriarchal authority by invoking male dominance to uphold traditional restrictions, creating multiple layers of gendered control. Framing dietary restrictions as protective care further legitimized patriarchal control, complicating resistance by presenting constraints as expressions of love rather than oppression.

#### Recommendations for addressing food avoidance

Rather than attempting to eliminate food taboos, interventions should negotiate incremental modifications that respect cultural rationales while enhancing nutrition. For example, programs might advocate for relaxing salt restrictions by day 3 rather than day 6 postpartum or develop eclipse-day nutrition strategies that work within ritual constraints while maintaining adequate intake. The Suaahara program’s social behavior change communication approach, which used culturally sensitive messaging through multiple channels, provides a model for gradually shifting harmful practices (71).

Engaging religious leaders is critical, particularly for addressing eclipse fasting and other ritual food restrictions. Co-creating interventions where Maulanas and Pandits endorse nutritionally beneficial practices as divinely sanctioned could leverage their authority to support maternal health. Practical strategies for compensating for foods that are avoided should be developed. If sour foods are restricted, alternative sources of vitamin C could be identified and promoted. Similarly, if “hot” foods like eggs face resistance, culturally acceptable protein alternatives could be emphasized.

Interventions aimed at improving maternal nutrition should incorporate strategies to engage male household members and mothers-in-law, thereby fostering awareness and dialogue about equitable household decision-making. The Mamata scheme’s approach of providing conditional cash transfers directly to women demonstrates how economic empowerment can shift household priorities toward maternal and child nutrition (72). Educational and community-based programs that explicitly address gender dynamics and economic dependency can empower women, reshape household practices, and enhance women’s practical control over dietary choices.

### Diversity in micronutrient consumption: cultural alignment and structural barriers

Our findings identify both promising alignments and concerning gaps in micronutrient consumption. The universal cultural endorsement of milk and the wide acceptance of bananas create natural entry points for nutrition promotion. Conversely, the variable acceptance of eggs, limited consumption of leafy greens, and challenges with IFA supplementation highlight areas requiring targeted intervention.

District variations in food patterns illustrate how local agricultural and market systems influence nutritional choices. The differential mention of fish, coconut water, eggs, and bananas between Nalanda and Samastipur highlights the need for nutrition programming to adapt to regional food systems and availability patterns.

The notable differences in micronutrient intake between ASHAs and mothers—where ASHAs are 3–4 times more likely to eat leafy greens and foods rich in vitamin A—indicate that knowledge alone does not ensure practice. Although ASHAs exhibit healthier dietary habits, obstacles such as the time required for food preparation (“who has time to clean so many leaves?”) and cultural beliefs (“hot” foods, such as eggs) remain, even among these trained health workers. The fact that only 45% of ASHAs completed IFA supplementation themselves, despite encouraging mothers to do so, highlights how structural and personal barriers hinder even well-informed practitioners from modeling best practices.

Pre-lacteal feeding and colostrum discarding are particularly troubling gaps between knowledge and practice, significantly impacting early infant nutrition. Our findings suggest that religious rituals often take precedence over biomedical advice, with infants receiving goat’s milk, water, or cow’s milk until priests deem it an auspicious time to initiate breastfeeding. Reports from traditional birth attendants about discarding colostrum and adding water during hot months—despite messages promoting exclusive breastfeeding—show how cultural authority and traditional beliefs can override health recommendations, even when ASHAs actively counsel otherwise.

#### Recommendations for enhancing diversity in micronutrient consumption

Nutrition interventions should leverage culturally endorsed foods, such as milk and bananas, as entry points, gradually introducing additional micronutrient sources within these accepted categories. For example, milk could be paired with micronutrient powders or fortified with additional nutrients while maintaining cultural acceptability. The multi-sectoral approach of Suaahara, which integrated nutrition with agriculture and health interventions, shows how dietary diversity can be promoted through complementary strategies (71).

For leafy greens, addressing preparation barriers is essential. Promoting batch-cooked spinach purée that can be stored and reused across meals could address the time constraints frequently mentioned by young mothers. Similarly, promoting locally available, culturally acceptable green vegetables with lower preparation requirements could increase consumption.

Iron-folic acid supplementation necessitates a multifaceted approach: streamlining distribution, implementing expiry tracking systems, addressing side effects through enhanced counseling, and empowering ASHAs with on-site hemoglobin testing to demonstrate effectiveness. Equipping ASHAs with proper storage containers and visual aids to explain the benefits of supplementation could enhance compliance.

For breastfeeding promotion, working with religious leaders to establish immediate initiation as compatible with traditional blessings could address the harmful gap between birth and breastfeeding commencement. Materials emphasizing colostrum as a “divinely designed first food” could reframe its value within traditional belief systems.

#### Enhancing ASHAs’ role in comprehensive nutrition promotion

Accredited Social Health Activists occupy a critical position as potential “cultural brokers” between biomedical and traditional knowledge systems (40, 41). However, their effectiveness can be significantly enhanced through targeted training, support, resource provision, and strategic collaboration with traditional health influencers. Nutrition education alone rarely changes diets. The Knowledge-to-Action framework helps explain why awareness must be adapted, implemented, and sustained in context (47, 48). ASHAs therefore need multifaceted, hands-on support beyond biomedical knowledge—such as cooking demonstrations, home visits, and regular follow-up—to help women translate guidance into daily food choices. Experience from Indian nutrition programs shows that information and resources, without strong delivery and uptake, result in limited dietary improvement (5). ASHA training should focus on developing cultural negotiation skills, including simplified, visually oriented counseling tools such as “food taboo negotiation toolkits” for addressing cultural practices like fasting and funeral attendance restrictions. ASHAs should be equipped with strategic compromise approaches that identify core nutrition principles to emphasize while temporarily accommodating cultural practices—for instance, ensuring colostrum consumption even when immediate breastfeeding conflicts with religious timing. Strategic collaboration with community influencers, particularly traditional midwives (Dais) and women’s self-help groups, can amplify ASHAs’ reach and cultural legitimacy, as these actors already possess established trust within communities and a deep understanding of local beliefs. Technology can further strengthen their capacity through mobile-based IFA distribution tracking with expiry alerts, digital counseling aids for promoting micronutrient-rich foods, and peer support networks that help them navigate the complex intersection of biomedical recommendations and cultural constraints, ultimately improving dietary practices among pregnant women they serve.

## Limitations and future directions

### Limitations

Several methodological constraints warrant consideration when interpreting our findings. The reliance on self-reported dietary practices introduces potential recall and social desirability biases, with ethnographic observations providing limited validation due to their restricted scope and duration. This cross-sectional design, although suitable for examining associations between sociocultural factors and dietary practices, precludes the establishment of temporal relationships or causal pathways. Another limitation is the absence of nutritional biomarkers or clinical measures (such as hemoglobin levels or micronutrient biomarkers) to corroborate self-reported intake, preventing confirmation of whether reported dietary differences translated into measurably different nutritional status.

We acknowledge that dietary practices may influence educational opportunities just as education influences dietary choices, and longitudinal studies are needed to disentangle these relationships. We acknowledge that ethnographic and FGD data from two districts (Nalanda and Samastipur) may not capture Bihar’s full cultural diversity. This limited qualitative scope requires cautious generalization of our cultural observations; future research across additional districts would help verify whether these patterns hold across Bihar’s diverse communities. While we examined several social determinants, including caste and education, we did not comprehensively assess economic status, land ownership, or household decision-making dynamics. The absence of male household members in our data collection represents another limitation. In rural India’s patriarchal structure, husbands and fathers-in-law often control household resources and influence food purchases. Our data from women may not fully capture the negotiation processes behind dietary decisions.

Additionally, our study did not capture information on Take-Home Rations (THR) from Anganwadi centers, precluding analysis of how government-provided supplementary nutrition may have influenced women’s diets. We recognize this as a scope limitation.

### Future directions

Future research should pursue several promising avenues to build upon this work. Longitudinal studies tracking women from pre-pregnancy through postpartum would provide deeper insights into dietary transitions and factors predicting sustained behavioral change. Future research should incorporate biomedical measures, for instance, tracking hemoglobin or other nutritional biomarkers, to objectively validate dietary intake and nutritional status. Such longitudinal studies could confirm whether reported diet changes translate into improved health outcomes. Testing culturally tailored interventions based on our findings could leverage areas of cultural alignment while addressing household power dynamics. Piloting nutrition interventions co-designed with local mothers, mothers-in-law, and ASHAs may enhance both cultural acceptability and effectiveness. For example, community workshops could help identify locally feasible diet modifications, which can then be tested for impact. Research examining how organizational support systems affect ASHAs’ ability to model and promote optimal nutrition practices would strengthen their effectiveness as cultural brokers. Engaging male household members, particularly husbands, and fathers-in-law, could reveal additional pathways for improving maternal nutrition. Studies of local food systems should assess how seasonal availability, market access, and climate change impact the availability and resilience of nutrient-dense foods in rural Bihar. Complementing self-reported practices with biomarker assessment would provide physiological validation and help target interventions more precisely. Finally, testing alternative training approaches for ASHAs based on the Knowledge-to-Action framework could enhance their practical skills for navigating cultural constraints rather than focusing solely on knowledge transfer.

## Conclusion

This study reveals the complex interplay between biomedical recommendations and cultural practices shaping perinatal nutrition in rural Bihar. Our comparative analysis of ASHAs and mothers identified persistent gaps in translating dietary guidelines into practice while highlighting culturally acceptable entry points for interventions.

Our study highlights several key insights. Education consistently influences dietary practices, though this relationship is nuanced—educated women increase overall intake yet selectively avoid certain foods. Structural barriers also affect knowledgeable practitioners like ASHAs, who often struggle to enhance their own dietary practices during pregnancy. Cultural consensus around rice-ghee combinations and milk consumption provides valuable opportunities for nutrition promotion. Additionally, household power dynamics, particularly the influential role of mothers-in-law as dietary gatekeepers, profoundly shape women’s food choices, regardless of individual knowledge. Moreover, discrepancies between ASHAs’ professional advice and their personal dietary behaviors undermine their effectiveness as role models.

These findings challenge simplistic approaches to maternal nutrition that focus solely on information dissemination. Effective interventions must work within cultural frameworks rather than against them, engaging key decision-makers like mothers-in-law and religious leaders while providing structural support to enable behavior change. Community health workers require more than knowledge transfer—they need practical tools for cultural negotiation, resolution of their dual role conflicts, and systemic support to model recommended practices.

Breaking the cycle of maternal undernutrition in low and middle-income regions demands interventions that respect cultural logic while creating feasible pathways for dietary improvement. Success lies not in replacing traditional practices but in identifying where biomedical and cultural wisdom align and then building upon these points of convergence.

## Data Availability

The datasets presented in this study can be found in online repositories. The names of the repository/repositories and accession number(s) can be found below: https://osf.io/7hakw/?view_only=cbdcf2a0403e40cc92121d00f373b4d0.
