# Supplementary material for: From counsel to consumption: examining sociocultural influences on perinatal nutrition in rural India
Source: Front Nutr. 2025 Aug 7;12:1645528. doi: 10.3389/fnut.2025.1645528 (PMC12367784; doi:10.3389/fnut.2025.1645528)
Supplement: Supplementary file 1 [file Data_Sheet_1.DOCX]

Comprehensive Narrative Report
Ethnographic Observations (Ethno)– Perinatal Diet Practices, Bihar

# Executive Summary

This report synthesises three weeks of shadowing Accredited Social Health Activists (ASHAs) and allied stakeholders. Ethnographic field notes illuminate how biomedical recommendations intersect—or clash—with entrenched ritual norms in the postpartum period. Key findings echo and enrich the earlier FGD + KII analysis: caloric intake is front‑loaded with sweets and milk, food taboos delay balanced meals, micronutrient supplementation falters amid mistrust, and frontline workers navigate dilapidated facilities and erratic incentives.

# 1  Methods

• Field team shadowed 13 ASHAs across four blocks, attended two PHC ‘family‑planning days’, and carried out participant observation in seven households during HBNC visits.

• Supplemented by informal interviews with one ASHA trainer, two ANMs, one dai, and multiple family members.

• Data recorded as detailed narrative notes; thematic coding followed the three dietary domains plus service‑delivery context.

# 2  Findings

## 2.1  Caloric adequacy after birth

Sweet, energy‑dense foods dominate the first postpartum week; grains and savoury items are deferred until the chhatti ceremony.

- “I am on a diet of milk and ginger halwa … I cannot have any grains before the sixth day.” – New motherciteturn1file4
- “Women were given sweets (halwa) and milk for the next six days.” – ASHA recounting her own deliveriesciteturn2file2

Hospital meals (rice, dal, chicken) are routinely refused as ‘heavy’ or ritually impure.

## 2.2  Food avoidance & ritual observance

- “A new mother does not eat salt … cannot touch the hand‑pump for 42 days.” – Village elderciteturn1file16
- “She will stay in that area till the sixth day and will be fed only milk and sweets.” – Maternal grandmotherciteturn1file14

Fire bowls for massage, bans on bathing, and mobility restrictions persist, though some ASHAs advise sponge baths for under‑weight infants.

## 2.3  Micronutrient intake & supplementation

- “I threw away all the IFA tablets – they made me nauseous.” – Pregnant womanciteturn1file4
- “I was given tablets but they were past expiry; I haven’t started yet.” – 5‑month‑pregnant womanciteturn1file0

Milk and banana remain acceptable; eggs are absent. Expired or mistrusted supplements and lack of ANM follow‑up erode coverage.

## 2.4  Pre‑lacteal feeding & breastfeeding

- “The child is fed goat’s milk until the pandit gives the exact time to start breastfeeding.” – Dai’s husbandciteturn1file9
- “Newborns were given water or cow’s milk until a priest was consulted.” – Senior ASHAciteturn1file16

Religious timing often overrides immediate initiation of breastfeeding despite ASHA counselling.

## 2.5  Frontline service environment

- Delayed or partial payment: “We haven’t received RI allowances for 2016‑18; the accountant takes a 20 % cut.” – ASHAciteturn1file10
- Out‑of‑pocket coping: “Sometimes I spend ₹500‑1000 of my own money during a delivery.” – ASHAciteturn2file15
- Facility shortages: “There is no drinking‑water facility at the PHC for the last four years.” – ASHAciteturn2file0
- Informal fees: “ASHAs also charge ₹200 but most of it goes to PHC staff.” – ASHA trainerciteturn1file2

# 3  Alignment and Divergence with FGD/KII Findings

Ethnographic observation corroborates FGD/KII themes while exposing on‑the‑ground nuance:

- Calorie boost via halwa & milk echoed across all data sources.
- Pre‑lacteal goat/cow milk uncovered only through shadowing; FGDs under‑reported this practice.
- ASHAs’ tactical spending and queue‑skipping deepen understanding of incentive shortfalls noted in KIIs.

# 4  Programme Implications

- Co‑design postpartum meal plans that pivot from day‑6 rituals toward gradual inclusion of grains & vegetables.
- Streamline IFA distribution and expiry‑tracking; empower ASHAs with on‑site hemoglobin testing kits.
- Provide PHCs with basic amenities (potable water, ASHA rest areas) to bolster trust and compliance.
- Leverage proven Mobile Kunji messages but localise audio on goat‑milk myths and early breastfeeding.
