# Supplementary material for: From counsel to consumption: examining sociocultural influences on perinatal nutrition in rural India
Source: Front Nutr. 2025 Aug 7;12:1645528. doi: 10.3389/fnut.2025.1645528 (PMC12367784; doi:10.3389/fnut.2025.1645528)
Supplement: Supplementary file 2 [file Data_Sheet_2.DOCX]

Comprehensive Narrative Report
Focus Group Discussions (FGDs) – Perinatal Diet Practices, Bihar

# Executive Summary

Eight focus‑group discussions (FGDs) with 78 participants (40 young mothers, 38 mothers‑in‑law) in Nalanda and Samastipur reveal how biomedical nutrition guidance is interpreted, reshaped, and sometimes resisted in household practice. Three domains structure the findings: (1) caloric adequacy, (2) food avoidance, and (3) micronutrient‑rich foods. Rice fortified with ghee is celebrated by both generations, while taboos on sour or ‘hot’ foods, salt withdrawal, and eclipse fasting restrict diet diversity. Milk, banana and coconut water are universally accepted “strength” foods; eggs gain traction with young mothers, whereas fish/meat remain elder‑driven and supply‑dependent. Interventions that amplify foods already culturally endorsed, and negotiate (rather than negate) restrictive taboos, are likely to achieve greater uptake.

# 1  Background

Bihar has among India’s highest burdens of maternal under‑nutrition. Accredited Social Health Activists (ASHAs) are trained to promote balanced diets, but elders—particularly mothers‑in‑law—retain authority over what pregnant and lactating women actually consume. Understanding the dialogue between biomedical messages and cultural logic is therefore crucial for programme design.

## Study Objectives

- Compare perinatal dietary practices between young mothers and mothers‑in‑law across three domains: caloric adequacy, food avoidance, and micronutrient‑rich foods.
- Identify alignment and gaps between cultural practices and biomedical recommendations.
- Explore district‑level and generational influences shaping food choice.

# 2  Methods

## 2.1 Setting and Participants

• Locations: Nalanda and Samastipur districts (rural blocks with comparable caste and livelihood profiles).

• Participants: 40 young mothers (<2 years postpartum) and 38 mothers‑in‑law selected through local Anganwadi registers.

## 2.2 Data Collection

Semi‑structured guides covered food beliefs, daily menus, pregnancy rituals, and decision‑making power. FGDs were audio‑recorded, transcribed verbatim in Hindi, and translated to English.

## 2.3 Analysis & Trustworthiness

- Rapid descriptive coding of each practice/belief statement.
- Inductive grouping into sub‑themes; cross‑checked with study objectives.
- Frequency tallies by speaker type and district to flag dominant voices.
- Peer debrief with two independent coders; discrepancies resolved by consensus.

# 3  Findings

## Theme 1 — Caloric Adequacy

*Why it matters: Adequate energy intake underpins maternal weight gain and postpartum recovery.*

### 1.1 Staple carbohydrates

Insight: Rice/khichri dominate meals; elders frame rice as “heavier” than wheat, especially in 3rd trimester.

**Illustrative quotes:**

“Rice keeps the stomach full; without it the mother feels empty.” [Mother‑in‑law]

“I asked for roti but they said only rice gives ‘weight’ to the baby.” [Young mother]

### 1.2 Energy‑dense additions

Insight: Ghee is the culturally sanctioned energy booster; affordability, not acceptability, limits use.

**Illustrative quotes:**

“One spoon of ghee in every plate—doctor or no doctor.” [Mother‑in‑law]

“If ghee is too costly we use mustard oil, but elders frown.” [Young mother]

### 1.3 Hydration & infant‑feeding work‑arounds

Insight: Elders routinely add water to breast‑feeds in hot months despite exclusive‑breast‑feeding messaging.

**Illustrative quotes:**

“In June heat the child needs pani along with milk, else he burns.” [Mother‑in‑law]

“ASHA says no water, but the baby cries; I give a spoon.” [Young mother]

## Theme 2 — Food Avoidance

*Why it matters: Taboos can narrow diet diversity during critical windows of foetal growth and maternal recovery.*

### 2.1 Taste/Temperature taboos

Insight: Sour & “hot” foods feared to cause miscarriage or body heat; rule enforced by elders.

**Illustrative quotes:**

“Sour cuts the womb like blade—better stay safe.” [Mother‑in‑law]

“I craved mango pickle but mother said ‘too garam’.” [Young mother]

### 2.2 Colour taboos

Insight: Black‑coloured foods linked to fears of a dark or weak baby.

**Illustrative quotes:**

“Black food makes the baby black and weak.” [Mother‑in‑law]

### 2.3 Ritual fasting / eclipse

Insight: During solar eclipse no food is prepared or eaten; mothers‑in‑law impose strict compliance.

**Illustrative quotes:**

“During the eclipse even a drop of water can curse the child.” [Mother‑in‑law]

“I hid a biscuit but felt guilty.” [Young mother]

### 2.4 Salt restriction

Insight: Salt withheld for the first 5–6 days postpartum to ‘purify milk’.

**Illustrative quotes:**

“Till Chhathi my tongue forgets salt; only then milk is sweet.” [Young mother]

## Theme 3 — Micronutrient‑Rich Foods

*Why it matters: Quality of calories determines maternal and foetal micronutrient status (iron, calcium, folate, protein).*

### 3.1 Dairy

Insight: Milk universally praised; buffalo milk preferred where available.

**Illustrative quotes:**

“A glass of buffalo milk makes bones solid like bamboo.” [Mother‑in‑law]

### 3.2 Fruits & natural drinks

Insight: Banana & coconut water seen as cooling and digestion‑friendly; supply differences visible by district.

**Illustrative quotes:**

“Two bananas settle the stomach and baby sleeps.” [Young mother]

“Coconut water cools the ‘fire’ inside.” [Mother‑in‑law]

### 3.3 Animal protein

Insight: Eggs gain traction with young mothers; elders split over ‘heat’ concerns. Fish/meat promoted by elders when affordable.

**Illustrative quotes:**

“Doctor says one egg a day, so I eat when nobody sees.” [Young mother]

“Fish grows the baby’s brain—river gift from God.” [Mother‑in‑law]

### 3.4 Green leafy vegetables

Insight: Low spontaneous mention; prep‑time cited as barrier.

**Illustrative quotes:**

“Palak is good, but who has time to clean so much leaf?” [Young mother]

## 3.4 District‑Specific Patterns

- Nalanda FGDs mention fish and coconut water twice as often as Samastipur, reflecting riverine markets.
- Samastipur groups reference eggs and bananas more frequently, mirroring local poultry and orchards.

## 3.5 Generational Power Dynamics

Mothers‑in‑law act as gate‑keepers of taboos and food distribution. Young mothers comply publicly but adopt hidden strategies (e.g., eating eggs privately) to integrate biomedical advice. Negotiation—not confrontation—is their coping mechanism.

# 4  Discussion

Findings echo earlier work on food taboos in South Asia, but add nuance on district supply chains and inter‑generational negotiation. When biomedical advice aligns with culturally accepted foods (e.g., milk, banana), adoption is seamless. Where misaligned (exclusive breastfeeding, leafy green promotion), compliance is partial or covert. Programme designers should therefore ‘scaffold’ new behaviours onto existing positives and frame taboo relaxation as incremental rather than oppositional.

## 4.1 Programme & Policy Implications

- Leverage Shared Positives – Use rice + ghee + banana as an entry‑point for balanced‑plate counselling.
- Negotiate, Don’t Nullify – Encourage phased relaxation of taboos (e.g., salt from Day 3) instead of outright bans.
- Elder‑Focused Messaging – Develop materials that position mothers‑in‑law as guardians of ‘modern‑plus‑traditional’ nutrition.
- Reduce Preparation Burden – Promote batch‑cooked spinach purée that can be reused across meals, addressing time constraints.

## 4.2 Study Limitations

- FGDs represent perceptions, not actual intake; recall bias possible.
- District sample limited to two blocks; findings may not generalise to northern Bihar.
- Caste, religion, and education were not uniformly captured, limiting social‑determinant analyses.

# 5  Conclusion

Young mothers and mothers‑in‑law share the goal of safeguarding maternal and newborn health. Interventions that respect cultural logics, while gently negotiating restrictive practices, stand the best chance of translating knowledge into daily diets.

# Appendix  | Frequency of Statements by Theme & Speaker

| Dietary Domain | Mother‑in‑law | Other | Young mother |
| --- | --- | --- | --- |
| Caloric adequacy | 53 | 5 | 34 |
| Food avoidance | 134 | 0 | 94 |
| Micronutrient‑rich foods | 170 | 6 | 130 |
| Other | 545 | 7 | 463 |
