# Supplementary material for: From counsel to consumption: examining sociocultural influences on perinatal nutrition in rural India
Source: Front Nutr. 2025 Aug 7;12:1645528. doi: 10.3389/fnut.2025.1645528 (PMC12367784; doi:10.3389/fnut.2025.1645528)
Supplement: Supplementary file 3 [file Data_Sheet_3.DOCX]

Comprehensive Narrative Report
Key‑Informant Interviews (KIIs)– Perinatal Diet Practices, Bihar

# Executive Summary

Key‑informant interviews with six stakeholder cadres (ASHAs, Anganwadi Workers, Dais, Rural Medical Practitioners, and Muslim/Hindu religious leaders) produced 1 279 coded statements on perinatal diet. Three domains structure the findings—caloric adequacy, food avoidance, and micronutrient‑rich foods—mirroring the focus‑group synthesis. Consensus spans staples (rice/khichdi), ghee fortification, and milk/banana as ‘strength foods’. Gaps surface around eggs, IFA tablets, and eclipse fasting, underscoring the need for cadre‑specific messaging and religious‑leader engagement.

# 1  Methods

• Sample: 410 ASHAs, 236 AWWs, 354 Dais, 118 RMPs, 69 Mulanas, 92 Pandits (statements).

• Data: Semi‑structured KIIs using a guide parallel to FGD prompts; recorded, transcribed, translated.

• Analysis: Rapid coding → domain grouping → frequency counts → quote selection for salience and diversity.

# 2  Findings

## Caloric Adequacy

*Why it matters: See sub‑themes.*

### 1.1 Staple carbohydrates

Insight: Rice/khichdi constitute the baseline meal; Dais and ASHAs urge an extra serving in the third trimester.

**Illustrative quotes:**

“After delivery Mother is given Bread, Biscuit, Milk, Rice and Vegies to eat” [AWW]

“fish, meat, pulses and rice are cooked and mother take this food by taking child on lap” [ASHA]

### 1.2 Energy‑dense additions

Insight: Ghee remains the culturally preferred calorie booster; AWWs promote, RMPs note cost barriers.

**Illustrative quotes:**

“Ghee with bread” [Dai]

“Halwa made up of Rice, Ginger, Turmeric, Ghee and jaggery is given to mother so that her body remains warm” [Dai]

### 1.3 Hydration tweaks

Insight: Leftover rice water (*maad*) and sweet tea emerge as low‑cost energy drinks, mainly from Dais.

**Illustrative quotes:**

“Ghee with Tea” [Dai]

“Tea” [Pandit]

## Food Avoidance

*Why it matters: See sub‑themes.*

### 2.1 Mobility restrictions

Insight: All cadres reiterate no travelling or heavy lifting late in pregnancy, citing miscarriage risk.

**Illustrative quotes:**

“Woman should avoid travelling” [RMP]

“women should avoid going out of the house and travelling” [ASHA]

### 2.2 Ritual & eclipse taboos

Insight: Religious leaders emphasise eclipse fasting; ASHAs offer mitigation advice (ORS, meal timing).

**Illustrative quotes:**

“During Solar eclips, a thread/ wood equivalent to the height of the pregnant woman is hanged on the wall” [Dai]

“Mother and child are bathed. A thread is tied around the waist of the child.Kohl is applied” [AWW]

### 2.3 Contact taboos

Insight: Avoiding funerals/dead bodies to ward off ‘evil influence’ is endorsed across groups.

**Illustrative quotes:**

“Pregnant woman should avoid looking at dead bodies/funerals” [AWW]

“Pregnant woman should avoid looking at dead bodies if she see's she should see till the end” [Dai]

## Micronutrient‑Rich Foods

*Why it matters: See sub‑themes.*

### 3.1 Milk & banana

Insight: Milk tops every informant list; bananas ranked second as easy, ‘cool’ snack.

**Illustrative quotes:**

“After delivery Mother is given Bread, Biscuit, Milk, Rice and Vegies to eat” [AWW]

“Colustrum is thrown and then mother milk is feeded to child” [Dai]

### 3.2 Protein foods

Insight: Egg promotion led by ASHAs; fish/meat endorsed by Mulanas & Pandits post‑delivery.

**Illustrative quotes:**

“AKIKA should be performed after 6 days of delivery.in case of boy child a 2 goats meat should be distributed.in case of girl child 1 goat meat should be distributed.” [Mulana]

“Meat” [ASHA]

“Egg” [ASHA]

### 3.3 Leafy greens & supplements

Insight: Spinach and IFA tablets championed by frontline workers; elders mention greens without dosage clarity.

**Illustrative quotes:**

“BP test,TT,Weight,IFA on AWC” [ASHA]

“Spinach” [Dai]

# 3  Alignment & Gaps Across Cadres

Rice/khichdi + ghee and milk/banana enjoy multi‑stakeholder agreement; however, eggs and IFA tablets are still debated. ASHA and AWW narratives emphasise biomedical supplements, while religious leaders frame fish/meat as divinely sanctioned. Mobility and eclipse taboos persist despite ASHA mitigation advice.

# 4  Programme Implications

- Co‑create sermon scripts where Mulanas and Pandits endorse eggs & IFA as “gifts of God”.
- Develop low‑cost ghee‑swap counselling cards for RMPs to distribute.
- Compose a ‘Taboo negotiation toolkit’ (eclipse fasting, funeral attendance) for ASHAs.
- Use rice‑ghee‑banana visual plate as common branding across all cadres.

# 5  Limitations

- Statements reflect informant recommendations, not direct observation of household behaviour.
- Cadre counts unequal; ASHA statements dominate quantitative tallies.
- Some religious leaders provided brief interviews yielding fewer diet details.

# 6  Conclusion

Stakeholder voices confirm entrenched cultural logic around perinatal diets. Leveraging shared positives while addressing cadre‑specific gaps—particularly eggs, IFA tablets, and restrictive taboos—offers a feasible path to improved maternal nutrition in Bihar.

# Appendix  | Statement Frequency by Domain & Cadre

| Domain | ASHA | AWW | Dai | Mulana | Pandit | RMP |
| --- | --- | --- | --- | --- | --- | --- |
| Caloric adequacy | 23 | 8 | 24 | 0 | 2 | 4 |
| Food avoidance | 30 | 20 | 41 | 10 | 20 | 12 |
| Micronutrient-rich foods | 98 | 45 | 58 | 6 | 10 | 21 |
| Other | 259 | 163 | 231 | 53 | 60 | 81 |
